# Headache and musculoskeletal pain in school children are associated with uncorrected vision problems and need for glasses: a case–control study

**DOI:** 10.1038/s41598-021-81497-w

**Published:** 2021-01-22

**Authors:** Hanne-Mari Schiøtz Thorud, Rakel Aurjord, Helle K. Falkenberg

**Affiliations:** Department of Optometry, Radiography and Lighting Design, National Centre for Optics, Vision and Eye Care, University of South-Eastern Norway, Kongsberg, Norway

**Keywords:** Signs and symptoms, Health care, Disease prevention, Paediatrics, Public health

## Abstract

Musculoskeletal pain and headache are leading causes of years lived with disability, and an escalating problem in school children. Children spend increasingly more time reading and using digital screens, and increased near tasks intensify the workload on the precise coordination of the visual and head-stabilizing systems. Even minor vision problems can provoke headache and neck- and shoulder (pericranial) pain. This study investigated the association between headaches, pericranial tenderness, vision problems, and the need for glasses in children. An eye and physical examination was performed in twenty 10–15 year old children presenting to the school health nurse with headache and pericranial pain (pain group), and twenty age-and-gender matched classmates (control group). The results showed that twice as many children in the pain group had uncorrected vision and needed glasses. Most children were hyperopic, and glasses were recommended mainly for near work. Headache and pericranial tenderness were significantly correlated to reduced binocular vision, reduced distance vision, and the need for new glasses. That uncorrected vision problems are related to upper body musculoskeletal symptoms and headache, indicate that all children with these symptoms should have a full eye examination to promote health and academic performance.

## Introduction

Headaches and neck and back pain are leading causes of years lived with disability globally, and the prevalence is gradually increasing from school age to early adulthood^1–5^. Further, the overall headache prevalence is increasing in children^3,6^, and research is needed to elucidate risk factors. One proposed risk factor is increased near work activities, and children spend increasingly more time performing near tasks, particularly using digital screens, both at school and in their spare time^7–9^. Near work (reading, screen use) requires precise coordination between the head-stabilizing muscles and the visual system. Vision problems have been identified as another risk factor for development of headaches and neck and back pain, inducing non-ergonomic static postures such as protruding head or asymmetrical neck postures^10–19^. Uncorrected vision problems (and need for glasses) substantially increase the load on the visual system and head-stabilizing muscles, provoking eyestrain, headache, and neck and back pain^10,20–28^. Even minor vision problems such as small refractive errors or reduced accommodation can cause these symptoms, as well as additional difficulty concentrating and poor coordination. These vision problems also challenge the ability to maintain good vision over time, and this may lead to unnecessary difficulties at school, lower academic performance, and risk for chronic pain development^17,29,30^. As such, good vision can be seen as an important health promoting factor and vision problems should be identified and corrected.

Headache frequency in children and adolescents seems to increase with the transition to adolescence (girls) and in relation to psychosocial stress and obesity^31,32^. Tension-type headache is the most prevalent headache in the general population and has a lifetime prevalence between 30 and 78% and a high socioeconomic impact^25,33^. The 1-year prevalence of headaches in children and adolescents has been shown to be 88%, and the overall prevalence of tension-type headache 58%. In a study published in 2015 including 493 Norwegian adolescents aged 12–18 years, the participants lost on average 9 days of activity per year because of headaches, significantly interfering with their daily activities and constituting a major health problem^34,35^.

Headaches and spinal pain are associated^36^. Knowledge regarding risk factors and interventions for musculoskeletal pain in children and adolescents is limited. Recent studies indicate associations between spinal pain and screen time, bad ergonomics, and socioeconomic and psychosocial factors, together with a higher pain prevalence in girls^4,5,37–41^. Headache and neck and back pain are personal burdens that reduce the quality of life, and identifying easily applicable and cost-effective interventions is important^42^. The aetiology is multifactorial, and treatments include medication, physical treatment, lifestyle modifications and psychological and cognitive-behavioural therapy^32,43^. Non-pharmacological treatments often involve extensive programs requiring high motivation^42^.

Treatment of uncorrected vision problems have received less attention in headache and neck and back pain management. Indications in the literature are that correcting vision problems (glasses/contact lenses) can treat headaches in children and adults^44,45^. Further, in Nordic school children, as many as 40% need glasses to obtain satisfactory vision during near tasks (book/tablet/computer) or far tasks (blackboard)^30,46–48^.

The purpose of this study was to describe possible associations between headache and neck, shoulder, and upper back pain and uncorrected vision, and elucidate risk factors in primary- and secondary-school children.

## Methods

### Subjects

The children in this study were recruited from Saltvern school (first to 10th grade), Bodø, Norway, during the period March to June 2017. Children who contacted the health nurses with unspecific pain in the neck/shoulders/upper back and/or headache and referred to a physiotherapist examination, were consecutively recruited to the pain group (n = 20). Of these 20 children, 11 (55%) reported neck, shoulder and/or upper back pain, 5 (25%) reported neck and/or upper back pain in combination with headache, and 4 (20%) reported headache. The mean age ± SD of the pain group was 11.7 ± 1.3 years (range 10–15 years, 11 girls and 9 boys). The control group was recruited from children who had not been in contact with the health nurse at Saltvern school. The control group was stratified regarding age and gender to match the pain group, with a mean ± SD age of 11.6 ± 1.4 years (range 10–13 years, 12 girls and 8 boys). In Norway, all schools are required to have an on-site health nurse service for all pupils^49^. At Saltvern school the health nurses are available each day between 8 and 15.30 h for scheduled and drop-in appointments.

Exclusion criteria for all participants were the following: Diagnosed or treated for headaches (including migraine) and neck/shoulder/back pain; diagnosed musculoskeletal disorders or systemic disease affecting eyes and the musculoskeletal system; previous injuries in head/neck/shoulders/back/eyes; daily use of medication affecting pain, blood circulation, vision and eyestrain; diagnosed and medicated attention-deficit/hyperactivity disorder (ADD/ADHD); diagnosed post-traumatic stress disorder; cognitive impairment; diagnosed dyslexia; impaired vision (decimal visual acuity < 0.3; LogMAR > 0.5), and non-fluent in Norwegian. Seven children in both groups had seasonal or perennial allergy. One child in the pain group and four children in the control group used glasses and/or contact lenses. In the control group, one child had classic Ehlers-Danlos syndrome (EDS)^50^, and one had asthma.

All participants and parents received verbal and written information about the study, and written informed consent was obtained from all children and parents. The study protocol was approved by the Norwegian Regional Committee for Medical and Health Research Ethics (2017/108) and followed the Declaration of Helsinki.

### Physical examination

A targeted examination procedure was developed for the purpose of this study based on validated tests^51–53^. The following variables were included: sitting posture (protruding head, asymmetrical neck), range of motion of the neck (rotation, lateral flexion, flexion; normal/decreased), range of motion of the shoulders (rotation, flexion; normal/decreased) and palpation of myofascial trigger points in musculature (breast, upper shoulders, interscapular; tender/not tender). All children were examined by one experienced physiotherapist who was blinded to group allocation.

### Eye examination

A comprehensive eye examination was performed according to international clinical guidelines^54,55^ by an experienced authorised optometrist (RA). All participants were examined in the same room with an ambient illumination of approximately 500 lx. The structured, age-appropriate patient history interview^54,55^ included vision problems during near and distance work (with and without current glasses/contact lenses), ocular and general health, medication, and family ocular and general health. Symptoms of headache, double vision, blurred vision at near/distance, moving/jumping letters, and photophobia were registered in relation to frequency, severity and duration, location (distance/near, frontal/temporal/suboccipital), onset in relation to time of day (early morning/during school/evening), and type of activity. A symptom was defined as positive if occurring ≥ 1 time/week for more than 3 months during the last year, with a moderate intensity and duration. Headache was further defined as an episode of pressing or tightening quality lasting more than 30 min and having a unilateral or bilateral location.

The eye examination included habitual and best-corrected logMAR visual acuity (6 m and 40 cm), dominant eye (near/distance), refractive error with and without cycloplegia (1–2 drops Cyclopentolate Minims 1%), cover test (near/distance), accommodative convergence/accommodation (AC/A) ratio (Howell phoria card), near point of convergence (NPC), monocular and binocular accommodation amplitude (AA) (RAF-rule)^56^, motility, confrontation visual field (Donders’ test), pupillary distance, colour vision, stereo acuity (TNO test), fundus examination, anterior chamber depth (Van Herick technique), intraocular pressures, vergence facility, and facility of accommodation (monocular/binocular). Additional binocular measurements were accommodative response (cross-cylinder card), near fusional reserves (positive/negative), accommodation reserves (positive/negative), and accommodation accuracy and lag (monocular estimate method (MEM) retinoscopy).

For analysis, spherical equivalent error (SER) was calculated in dioptres (D). Refractive errors were defined as myopia (SER ≤  − 0.50 D), emmetropia (− 0.50 D < SER <  + 0.50 D), mild hyperopia (≥ + 0.50 D, SER <  + 2.00 D), moderate to high hyperopia (SER ≥  + 2.00 D), astigmatism (≥ 0.75 DC) and anisometropia (difference between the eyes ≥ 1.00 D). Normal visual acuity was defined as best corrected VA ≤ 0.0 logMAR, orthophoria as 2 prism dioptres (pd.), exophoria to 1 pd. esophoria for distance, and 6 pd. exophoria to 0 pd. esophoria for near. Normal NPC was defined as ≤ 10 cm, AA as > 9 D, and AC/A ratio as 4:1 ± 2 pd. Normal values are in line with previous studies^46,48,57,58^. Glasses for uncorrected refractive errors were recommended when the child had (1) moderate to high hyperopia, (2) mild hyperopia and ≥ 2 positive symptoms, (3) myopia, (4) reduced binocular function and ≥ 2 positive symptoms, (5) best-corrected visual acuity improvement of − 0.1 logMAR or more, or a combination of these. In addition, children already wearing glasses were recommended new glasses if there was a change of ≥ 0.50 best corrected SER^54,55^. Children defined as emmetropic, with normal eye health and no symptoms, were not recommended glasses.

**Figure 1:**
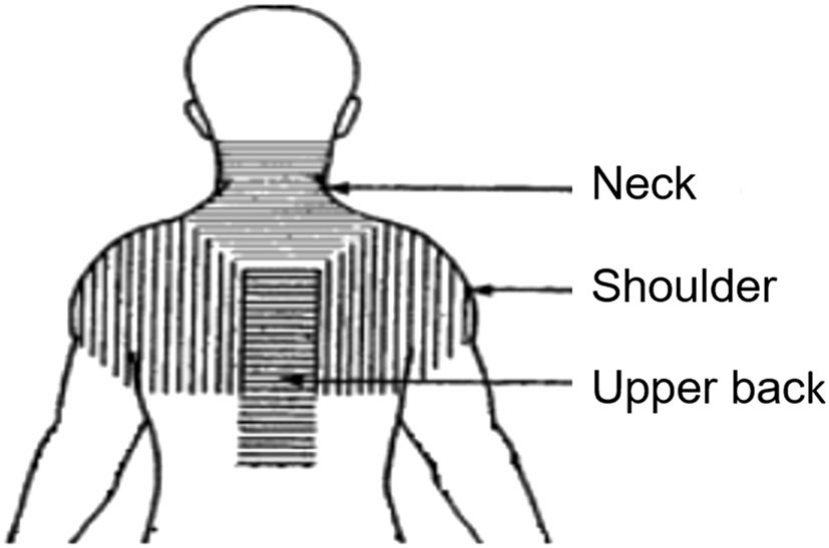
Localization of neck, shoulders and upper back. Reproduced by permission^92^.

In addition, physical activity (including organized activities, weekdays/days off), digital screen use (mobile phone, tablet, computer during weekdays/days off) and associated symptoms were answered using a structured interview questionnaire. Pain in the eyes/head/neck/shoulders/upper back during screen use (Fig. 1) were scored using the Wong-Baker FACES Pain Rating Scale^59^. This scale uses faces to help children communicate about their pain. Each of the six faces (0, 2, 4, 6, 8, or 10 points) represents a different facial expression of pain. Children were encouraged to describe their history and symptoms during the eye examination and the interview questionnaire by themselves. Parents/guardians were asked to elaborate and add information when necessary. To ensure maintained attention and avoid tiredness, the questionnaire was performed at a second visit within two weeks from the eye examination.

Efforts and attention were paid to avoid investigator bias during the eye examination regarding group allocation. However, due to the nature of an eye examination, and especially, history taking, it was not possible to ensure that the optometrist was completely blinded. As history taking and recording of symptoms were structured, and most vision tests are objective, the effect of potential bias were considered low, in line with previous findings^60^. The optometrist was not aware of the results from the physiotherapist at the time of the eye examination.

### Statistics

Raw data were assessed for normality using Q-Q plots and the Shapiro–Wilk test. Differences between the pain and control groups were tested by one-way analysis of variance (ANOVA) and independent-samples t-tests. A paired sample t-test was used for comparing dependent variables. Chi-square independence tests were used to evaluate associations between categorical variables. Pearson’s correlation coefficient (*r*) was used to investigate associations between continuous variables. Point-biserial correlations were run to determine the relationship between categorical and continuous variables. To protect from Type I errors, Bonferroni corrections were conducted for multiple comparisons when suitable^61^. Analysis and distributions using refractive errors included right eye only, as there were no significant differences between the right and left eyes (paired sample t-test, p > 0.05) for either group. A statistical difference was set at p < 0.05 (two-tailed). Statistical analyses were performed in IBM SPSS Statistics (Version 24, US).

## Results

### Physical examination

Trigger points and physical deviations were present in both groups (Table 1), and upper shoulder trigger points (pericranial tenderness) were the most common finding. The physical examination revealed that eighteen (90%) of the children in the pain group had upper shoulder trigger points, which was significantly more frequent compared to the 8 (40%) in the control group (χ^2^(1, n = 40) = 11.00, p = 0.001). There were no other significant differences between the groups.

**Table 1:** Frequency of trigger points and physical deviations.

Physical examination	Pain group (n = 20) n (%)	Control group (n = 20) n (%)
**Trigger points**		
Breast	9 (45)	3 (15)
Upper shoulder	18 (90)*	8 (40)
Interscapular	9 (45)	5 (25)
**Sitting posture**		
Protruding head	8 (40)	3 (15)
Asymmetrical neck	2 (10)	0 (0)
**Range of motion, deviations of the neck**		
Rotation	2 (10)	2 (10)
Lateral flexion	1 (5)	6 (30)
Flexion	4 (20)	3 (15)
**Range of motion, deviations of the shoulders**		
Rotation	0 (0)	0 (0)
Flexion	0 (0)	0 (0)

*Statistical significant difference between groups (Bonferroni adjusted alpha level of 0.005 (0.05/10)).

### Eye examination

All children had good ocular health and correctable vision. Table 2 shows that the average refractive error was mild hyperopia in both groups. Frequencies of refractive errors are shown in Table 3, and it can be seen that there was one child with myopia, one with anisometropia and two with astigmatism. The cycloplegic refraction, as expected, significantly increased hyperopia by approximately + 0.5D in both groups. There were no significant differences in mean refractive error between the groups (Table 2). Table 2 also shows that on average, the children had normal habitual and best corrected visual acuity (VA). In the pain group there was a significant improvement in best corrected VA in the right eye (n = 20, p = 0.023).

**Table 2:** Eye examination—average visual acuity, refractive error and binocular status.

	Pain group (n = 20) Mean ± SD	Control group (n = 20) Mean ± SD
**Habitual LogMAR VA (6 m)**		
RE	0.05 ± 0.12	0.01 ± 0.12
LE	− 0.02 ± 0.06	− 0.02 ± 0.07
BIN	− 0.09 ± 0.06	− 0.09 ± 0.05
**Best corrected logMAR VA (6 m)**		
RE	− 0.02 ± 0.06	− 0.02 ± 0.04
LE	− 0.02 ± 0.04	− 0.01 ± 0.05
BIN	− 0.07 ± 0.04	− 0.09 ± 0.04
**Cycloplegic best corrected logMAR VA (6 m)**		
RE	− 0.01 ± 0.04	0.02 ± 0,04
LE	− 0.01 ± 0.03	0.01 ± 0.03
BIN	− 0.05 ± 0.05	− 0.07 ± 0.05
**Retinoscopy (SER)**		
RE	0.93 ± 0.66^**#**^	0.96 ± 0.50^**#**^
LE	1.16 ± 0.62^**#**^	0.91 ± 0.44^**#**^
**Cycloplegic retinoscopy (SER)**		
RE	1.53 ± 1.00	1.51 ± 0.64
LE	1.66 ± 0.90	1.48 ± 0.62
**Best corrected refractive error (SER)**		
RE	0.59 ± 0.62^**#**^	0.63 ± 0.46^**#**^
LE	0.70 ± 0.54^**#**^	0.51 ± 0.32^**#**^
**Cycloplegic best corrected refractive error (SER)**		
RE	1.05 ± 0.90	1.18 ± 0.55
LE	1.18 ± 0.82	1.06 ± 0.58
**Binocular vision measurements**^**a**^		
AC/A	2.8 ± 1.2*	4.6 ± 3.3
NPC	7.5 ± 6.4	6.0 ± 1.4
TNO	75 ± 62	76 ± 96
VF	9.8 ± 4.5	8.7 ± 4.6
MEM, RE	1.80 ± 0.37	1.90 ± 0.42
MEM, LE	1.88 ± 0.39	1.88 ± 0.46
NRA	2.08 ± 0.43	1.99 ± 0.56
PRA	− 1.92 ± 2.28	− 2.29 ± 1.99
AA BIN	12.13 ± 5.99	15.33 ± 5.46

*VA* visual acuity, *SER* spherical equivalent error in diopters (D), *RE* right eye, *LE* left eye, *BIN* binocular (both eyes), *AC/A* accommodative convergence/accommodation ratio (dp,), *NPC* near point of convergence (cm), *TNO* TNO stereopsis (sec arc), *VF* vergence facility (number of cycles/min), *MEM* monocular estimate method retinoscopy (D), *NRA/PRA* negative/positive relative accommodation (D), *AA* amplitude of accommodation (D).

^a^One child in the pain group was excluded due to esotropia.

^#^Refraction: Statistically significant lower SER within group compared with cycloplegia at p < 0.05.

*Statistically significant difference between the groups at p < 0.05.

**Table 3:** Sample frequencies of reported symptoms, pain scores, vision results, and management.

	Pain group (*n* = 20) *n* (%)	Control group (*n* = 20) *n* (%)
**Symptoms; eye examination**		
Headache	16 (80)*	8 (40)
Frontal/temporal	9 (56)	5 (63)
Onset during school hours	13 (81)	7 (88)
Double vision	5 (25)	7 (35)
Blurred vision	7 (35)	6 (30)
Moving letters	4 (20)	5 (25)
Photophobia	10 (50)	6 (30)
≥ 2 symptoms	15 (75)*	7 (35)
**Symptoms during screen use; questionnaire**		
Eye pain	7 (35)	7 (35)
	1.2 ± 1.9	1.3 ± 2.0
Headache	13 (65)*	5 (25)
	2.5 ± 2.0*	0.8 ± 1.5
Neck pain	15 (75)	13 (65)
	2.7 ± 2.2	2.1 ± 2.2
Shoulder pain	8 (40)	6 (30)
	1.1 ± 1.5	1.1 ± 2.0
Upper back pain	11 (55)	7 (35)
	1.6 ± 1.7	1.2 ± 2.0
**Ametropia RE (cycloplegic SER, D)**		
Emmetropia (> − 0.50 D, < + 0.50 D)	4 (20)	1 (5)
Mild hyperopia (≥ + 0.50 D, < + 2.00 D)	13 (65)	17(85)
Moderate to high hyperopia (≥ + 2.00 D)	2 (10)	2 (10)
Myopia (≤ -0.50 D)	1 (5)	0 (0)
Anisometropia (≥ 1.00 D)	0 (0)	1 (5)
Astigmatism (≥ 0.75 DC)	2 (10)	0 (0)
**Hab VA RE**		
LogMAR (≤ 0.0)	10 (50)	12 (60)
LogMAR (≤ 0.1, > 0.0)	7 (35)	6 (30)
LogMAR (< 0.5, > 0.1)	3 (15)	2 (10)
**Best corrected VA RE**		
LogMAR (≤ 0.0)	19 (95)	20 (100)
LogMAR (≤ 0.1, > 0.0)	0 (0)	0 (0)
LogMAR (< 0.5, > 0.1)	1 (5)	0 (0)
**NPC (cm)**		
≤ 10	18 (90)	20 (100)
> 10 < 25	1 (5)	0 (0)
≥ 25	1 (5)	0 (0)
**AA ≤ 9 D**		
RE	9 (45)	4 (20)
LE	8 (40)	7 (35)
BIN	6 (30)	3 (15)
**AC/A**		
≤ 2.50	12 (60)	7 (35)
**Management**		
Recommended new glasses	15 (75)^**#**^	8 (40)^a^
Moderate to high hyperopia	2 (10)	1 (5)
Myopia	1 (5)	0 (0)
Mild hyperopia and ≥ 2 symptoms	3 (15)^b^	4 (20)^b^
Mild hyperopia, reduced AC/A and ≥ 2 symptoms	11 (55)	7 (35)

*SER* spherical equivalent error in diopters (D), *RE* right eye, *VA* visual acuity, *Hab* habitual, *NPC* near point of convergence (cm), *AA* amplitude of accommodation (D), *LE* Left eye, *BIN* binocular (both eyes), *AC/A* accommodative convergence/accommodation ratio (dp.). Symptoms during screen use are reported as frequencies and average pain scores (mean ± SD) (Wong-Baker FACES Pain Rating Scale^59^).

*Statistical significant difference between groups for symptoms (Bonferroni adjusted alpha level of 0.01 (0.05/5)).

^**#**^Statistical significant difference between groups (p < 0.05).

^a^Change in current glasses ≥ 0.75 D (n = 3).

^b^Also included in category below.

The frequencies of habitual and best corrected VA are shown in Table 3, and with the best corrected VA there was one child in the pain group that did not achieve normal vision due to known anisometropia.

Table 2 also shows that the pain group had a significantly lower accommodative convergence/accommodation ratio (AC/A) compared to the control group (one-way ANOVA (F(1,37) = 4.991, p = 0.032); independent-samples t-test (t(37) =  − 2.234, p = 0.032)). No other significant differences in mean values regarding binocular measurements were found between the groups. Although the binocular vision results on average were within normal limits in both groups (Table 2), Table 3 shows that two children in the pain group had reduced near point of convergence (NPC), 6 and 3 children had reduced binocular amplitude of accommodation (AA), and 12 and 7 had reduced AC/A in the pain and control group, respectively.

Table 3 shows the symptoms from the eye examination and the screen-use questionnaire. The results from the eye examinations showed that headache was significantly more frequent in the pain group (16/80%) compared to the in the control group (8/40%) (χ^2^(1, n = 40) = 6.67, p = 0.01). The location was mostly frontal or temporal, and onset was during school hours, but there was no significant difference related to location or onset. The eye examination revealed a significant increase of seven more children with headache in the pain group compared to the time of recruitment (χ^2^(1, n = 20) = 4.09, p = 0.043). Significantly more children in the pain group reported two or more symptoms during the eye examination compared to in the control group (χ^2^(1, n = 40) = 6.47, p = 0.01). Table 3 also shows that headache during screen use was more frequent in the pain group (χ^2^(1, n = 40) = 6.47, p = 0.01), and with a higher intensity score (one-way ANOVA (F(1,38) = 8.987, p = 0.005); independent-samples t-test (t(38) = 2.998, p = 0.005). The most frequent symptom during screen use was neck pain, present in approximately two-thirds of children in both groups. The screen-use questionnaire revealed that all the children regularly used a smart phone. In the pain group, 75% and 80% additionally used a tablet or a computer, respectively, compared to 90% and 75% in the control group. There were no significant differences regarding screen time or physical activity between the groups, and no significant correlations between reported symptoms and screen use. The total screen time was 3.4 ± 1.1 h (mean ± SD, range 1.0 to 5.5) during weekdays, which was significantly lower compared to 4.5 ± 1.7 h (mean ± SD, range 2.0 to 8.0) on days off (p < 0.000). The children were engaged in physical activity 3.0 ± 0.9 h (mean ± SD, range 1.5 to 5.5) on weekdays and 3.0 ± 1.2 h (mean ± SD, range 1.0 to 7.0) during days off.

Table 3 shows that new glasses were the main recommended treatment for all children. Significantly more children in the pain group were recommended new glasses (15), compared to the control group (8) (χ^2^(1, n = 40) = 5.01, p = 0.025). It can be seen that most children were recommended glasses for mild hyperopia, reduced AC/A, and ≥ 2 symptoms. New glasses was significantly associated with reduced binocular vision and habitual VA (AC/A ratio; *r* =  − 0.418, n = 39, p = 0.008/VA; *r* = 0.336, n = 40, p = 0.037). These children were explained to use their glasses for near work, to alleviate and prevent symptoms, and facilitate reading, writing, and screen viewing. Three children had moderate to high hyperopia and were recommended glasses for continuous use. The one child with myopia was recommended glasses mainly for distance use. Three of the children in the control group who already wore glasses, were recommended a change in prescription due to increased hyperopia.

### Associations between headache, trigger points and uncorrected vision

For all children, headache was significantly related to recommendation of new glasses (χ^2^(1, n = 40) = 11.53, p = 0.001). In addition, the presence and intensity of headache during screen use was significantly correlated with recommendation for new glasses (χ^2^(1, n = 40) = 5.51, p = 0.019; *r* = 0.366, n = 40, p = 0.020). The presence of both headache/headache during screen use and trigger points was also significantly associated with the need for new glasses (χ^2^(1, n = 40) = 5.01, p = 0.025/(χ^2^(1, n = 40) = 4.97, p = 0.026). Further, symptoms were related to reduced binocular vision and VA. Reduced binocular AA was associated with headache (*r* = – − 0.492, n = 40, p = 0.001) and combined headache and trigger points (borderline significant; *r* =  − 0.311, n = 40, p = 0.054). Increased presence of upper shoulder trigger points was significantly correlated with reduced habitual VA (RE: *r* = 0.341, n = 40, p = 0.031; LE: *r* = 0.356, n = 40, p = 0.024).

Overall, headache recorded during eye examination was associated with upper shoulder trigger points (χ^2^(1, n = 40) = 8.86, p = 0.003). The number of children with both headache and trigger points were significantly higher in the pain group (15/75%) compared to the control group (5/25%) (χ^2^(1, n = 40) = 10.00, p = 0.002). Also, headache during screen use was significantly related to the presence of trigger points in the pain group (χ^2^(1, n = 20) = 4.13, p = 0.042; *r* = 0.454, n = 20, p = 0.044). Significantly more children in the pain group (13/65%) had both headache during screen use and trigger points, compared to in the control group (2/10%) (χ^2^(1, n = 40) = 12.91, p = 0.000).

## Discussion

The results of this study showed that school children presenting with headaches and musculoskeletal pain also had uncorrected vision problems and needed glasses. The prescribed glasses were mainly for near work, such as reading/writing and screen use. Headache and trigger points were correlated to reduced binocular vision, lower VA and the need for new glasses. This suggests a relationship in children between upper body musculoskeletal symptoms, headache and the need for an optical correction. More children in the pain group were recommended new glasses due to hyperopia and reduced binocular vision compared to the control group. This is in line with a recent study investigating 7 to 15 year old children with uncorrected vision problems, where glasses were the main recommended treatment due to headaches, hyperopia, near vision problems, and reduced distance vision^48^. Only a few previous studies have examined headaches in relation to reduced vision, and the results indicate a relation between headaches and refractive errors^44,45,48,62–65^. However, only two of the studies used cycloplegic eye drops as recommended for evaluating refractive errors in children and young adults^66,67^, as such, headache in relation to mild hyperopia might have been ignored. Further, uncorrected vision also includes reduced visual functions, such as anomalies in visual acuity and binocular vision. Refractive error is the only risk factor mentioned in the international guidelines on classification of headaches^33^. Both reduced visual acuity and binocular vision should also be considered risk factors for headache and pericranial pain, as they pose an increased load on the visual system and head-stabilizing muscles, especially during near work^10,20–28,68^, in agreement with the present study.

More than twice as many children in the pain group (75%) had combined headaches and upper shoulder trigger points compared to the control group (25%), and headache was significantly associated with upper shoulder trigger points. Associations between headache and pericranial tenderness/trigger points in the neck and shoulder musculature have previously been shown in adults^69–72^. In children, knowledge of this association is limited, but it has been reported that approximately 20% of children with chronic tension-type headache also had pericranial trigger points^73,74^, which is lower than in this study. One reason for this difference could be that our study sample is older, and the presence of trigger points have been shown to increase with age^75^. Another reason could be type of headache. The eye examination in this study was performed according to international clinical guidelines^54,55^, however, the symptom registration was not detailed enough to be able to completely diagnose type of headache according to the International Classification of Headache Disorders^33^. The reported headaches were significantly associated with upper shoulder trigger points and had no significant correlations with reported photophobia. This may possibly point towards the definition of frequent episodic tension-type headache associated with pericranial tenderness (IHS, ICHD-3, 2.2.1)^33^. Increased pericranial tenderness is the most significant abnormal finding in patients with any type of tension-type headache, and the underlying pathophysiology is described as peripheral nociception from percranial and cervical musculature and central sensitization and increased excitability of the CNS^33,72^.

This study shows that headache is a complex symptom to understand, both for the individual and for health personnel, and that headache is often combined with both trigger points and neck and shoulder pain, in line with guidelines^32^. That more children in the pain group reported headaches to the optometrist than to the health nurse is an example of headache being difficult to understand. One explanation for this is that headache symptoms may be understood by the child as neck, shoulder or upper back pain. This is supported by findings that tension-type headache symptoms can extend into the neck and shoulders^72^. The eye and physical examination revealed that headaches and trigger points were also common in the control group, however, these children had not been in contact with health care personnel. Further, the results showed associations between symptoms and need for new glasses in 40% of the control group, in line with previous studies showing that 10–40% of school children need glasses to obtain satisfactory vision^30,46–48^. That the eye examination also revealed symptoms in the control group, supports that children often are unaware of symptoms of uncorrected vision problems. Particularly mild hyperopia, near vision problems and headaches may be difficult for a child to understand and complain about, and are often only identified by a thorough eye examination, when tested or asked specifically. It is a risk that the children found to have vision problems in this study, would have remained undiagnosed and uncorrected^30,46–48^. It is well known that children may have difficulties expressing and understanding symptoms of pain and discomfort, which our study also indicates^76^.

The children’s access to screens (mobile phone, tablet, computer) in this study was high, in accordance with national and international reports^7–9,77,78^. In both groups, eye pain, headache and neck/shoulder/upper back pain were all frequently reported screen-related symptoms, with neck pain reported in as many as two-thirds of the children. This is in line with previous literature, showing that digital screen-use may provoke headache, eyestrain, and upper body musculoskeletal pain in children and adolescents^41^. The severity of symptoms have been shown to increase with static non-ergonomic postures, vision problems and prolonged viewing time^16,20,21,26–28,41,79–83^. However, no significant differences in screen use or physical activity were found between the groups in this study. This was not unlikely, as the two groups were recruited from the same school and were balanced with regard to age and gender. The findings of increased headache and musculoskeletal pain in the pain group may therefore be due to other factors, such as uncorrected vision problems. Uncorrected vision and the need for glasses substantially increase the load on the visual system and head-stabilizing muscles, provoking symptoms such as neck pain and headache. Therefore, for example, near work such as screen watching will induce more symptoms in children who need glasses compared to children with normal vision^10,20–28^. Headaches and musculoskeletal symptoms in children have been shown to be associated with psychosocial stress and socioeconomic aspects^31,39^. However, these issues were not investigated in our study, and therefore the prevalence and possible effects on symptoms remain unknown.

### Vision status

The majority of children in this study had good habitual vision, and all but one in the pain group, achieved best corrected visual acuity of 0.0 logMAR (1.0 decimal acuity). Cycloplegic refraction showed that the most prevalent refractive error was low hyperopia, followed by emmetropia. Only one child was myopic. This is in line with other Nordic studies in children and adolescents^46–48,84^. Although most children had good binocular vision, the children in the pain group had a significantly reduced AC/A compared to the control group. The AC/A is indicative of the visual focusing ability, and is necessary to keep a target in focus over time, and when changing focusing distances (e.g. from near, when reading, to distance when looking at the blackboard). Symptoms of reduced AC/A are headaches, blurred vision, eyestrain, intermittent double vision and learning difficulties^47,85–87^. Low AC/A may also indicate weakness in the eye’s ability to converge and convergence insufficiency (CI)^88^. CI is one of the most common binocular dysfunctions^86^. All children with suspected CI were recommended glasses for all types of near work^58^. In the pain group, there was a significant correlation between headaches, trigger points and reduced binocular AA. That reduced accommodation is related to headache and pericranial tenderness can be explained by increased activity in the trapezius muscle, as more eye and neck stabilization is necessary to compensate for different visual demands when focusing at different distances^89^. It is also possible that the correlation between symptoms and reduced binocular vision, is caused by a direct link between the ciliary muscle/accommodation and the neck- and shoulder area^28^. Although this study was not able to answer a causational relationship between uncorrected vision problems and symptoms of headache and pericranial tenderness, the presence of significant associations, particularly in the pain group, indicate that vision problems should be identified and corrected to promote health and academic quality of life.

## Strengths and limitations

Even though the present study had a relatively small sample size, a strength is that the age-range is quite narrow and there is a gender balance in the sample, and thus the results may be representative for 10–15 year old school children. However, the results must be reproduced in larger, longitudinal studies to draw more solid conclusions. Another strength is that this study is one of few investigating and showing associations between symptoms of headaches, musculoskeletal complaints and vision problems in school children, supporting that uncorrected vision may be a risk factor. Due to the nature of an eye examination, and especially history taking, it was not possible to ensure that the optometrist was completely blinded. However, the history taking and recording of symptoms were structured, and most vision tests are objective, so the effect of potential bias were considered low in line with previous findings^60^. The physiotherapist was blinded to group allocation, and the optometrist was not aware of the results from the physiotherapist at the time of the eye examination. Future studies should also include psychosocial variables, such as stress and quality of life, known to be associated with the experience of pain.

## Conclusions

School children presenting with headaches and musculoskeletal pain have uncorrected vision and an increased need for near work glasses (e.g. reading, writing, screen use). Most children were hyperopic, supporting findings from other Nordic studies. Together with the increasing amount of daily near work such as screen work, this study highlights and supports the importance of regular eye examinations to prevent unnecessary symptoms and difficulties^7–9,48,90^. Importantly, this study shows that many school children, parents and teachers are unaware of symptoms of vision problems that may influence academic performance and quality of life^30,48^. Symptoms of headaches and the need for glasses are difficult to reveal in school children, and awareness should be raised in parents, teachers and school health services, as solutions may be easily available and important for quality of life. At present, reduced vision as a risk factor for headaches and pericranial pain is almost absent and incompletely described in guidelines, presumably caused by the low volume of research and lack of high-quality evidence^32,33,91^. Our study has elucidated the role of optimizing vision with glasses in prevention and treatment of musculoskeletal symptoms and headache, and highlights the need for more research.

## Data Availability

The datasets analysed during the current study are available from corresponding author upon reasonable request.

## Abbreviations

AA: Amplitude of accommodation (D)
AC/A: Accommodative convergence/accommodation ratio
BIN: Binocular (both eyes)
D: Diopter
LE: Left eye
MEM: Monocular estimate method retinoscopy
NPC: Near point of convergence
NRA/PRA: Negative/positive relative accommodation
RE: Right eye
SER: Spherical equivalent error
TNO: TNO stereopsis
VA: Visual acuity
VF: Vergence facility
